# A Novel Fabrication Method for Functionally Graded Materials under Centrifugal Force: The *Centrifugal Mixed-Powder Method*

**DOI:** 10.3390/ma2042510

**Published:** 2009-12-23

**Authors:** Yoshimi Watanabe, Yoshifumi Inaguma, Hisashi Sato, Eri Miura-Fujiwara

**Affiliations:** 1 Department of Engineering Physics, Electronics and Mechanics, Graduate School of Engineering, Nagoya Institute of Technology, Gokiso-cho, Showa-ku, Nagoya 466-8555, Japan; E-Mails: chw16515@stn.nitech.ac.jp (Y.I.); sato.hisashi@ nitech.ac.jp (H.S.); 2 Functionally Graded Materials Laboratory, Nagoya Institute of Technology, Gokiso-cho, Showa-ku, Nagoya 466-8555, Japan; E-Mail: emiura@nitech.ac.jp (E.M.-F.)

**Keywords:** Functionally Graded Material (FGM), centrifugal mixed-powder method, nano-particle, grindstone

## Abstract

One of the fabrication methods for functionally graded materials (FGMs) is a centrifugal solid-particle method, which is an application of the centrifugal casting technique. However, it is the difficult to fabricate FGMs containing nano-particles by the centrifugal solid-particle method. Recently, we proposed a novel fabrication method, which we have named the centrifugal mixed-powder method, by which we can obtain FGMs containing nano-particles. Using this processing method, Cu-based FGMs containing SiC particles and Al-based FGMs containing TiO_2_ nano-particles on their surfaces have been fabricated. In this article, the microstructure and mechanical property of Cu/SiC and Al/TiO_2_ FGMs, fabricated by the centrifugal mixed-powder method are reviewed.

## 1. Introduction

Functionally graded materials (FGMs) provide a reasonable compromise in terms of the properties of materials that would not be achieved otherwise. This is because the microstructure of the FGM is inhomogeneous and changing continuously in space. Thus, an FGM has a wide range of engineering applications. A wide variety of available processes have been reported for FGM fabrication, such as plasma spraying, powder metallurgy, physical vapor deposition (PVD), chemical vapor deposition (CVD), and so on [1,2,3]. For example, MoSi_2_/Al_2_O_3_ FGM with alumina contents varying from 20 to 80 mol % have been fabricated using a combination of tape casting and self-propagating high-temperature synthesis (SHS) [4]. Fabrication of FGMs from elemental powders of Ti and B by SHS technique was investigated by Cirakoglu *et al.* [5]. 

The centrifugal casting method is the casting process specifically adapted to the production of cylindrical parts without using a core. The molten metal is poured into a spinning mold and the spinning continued until the metal solidifies. The principal advantage of the centrifugal casting method is good mold filling combined with good microstructural control, which usually results in excellent mechanical properties [6]. In general the segregation caused by the difference of density between the particle and the melt is supposed to be avoided from the viewpoint of homogeneity. However, seen from a different angle, it is possible to create a compositional gradient utilizing this difference in material density [7,8,9]. This is the typical concept of a centrifugal method. The compositional gradient is then achieved primarily by the difference in the centrifugal force produced by the difference in density between the molten metal and solid particles.

The fabrication of the FGMs by the centrifugal method can be classified into two categories: the centrifugal solid-particle method and the centrifugal *in situ* method [10,11,12]. In the case of the centrifugal solid-particle method, the dispersed phase remains solid in a liquid matrix during the centrifugal casting. On the other hand, if the centrifugal force is applied during the solidification of both the dispersed phase and the matrix we have the centrifugal *in situ* method. 

In this review article, we focus on a centrifugal method [7,8,9,10,11,12] that can be of more practical interest. We will first briefly describe the centrifugal solid-particle method [7,8,13], and then explain our novel FGM fabrication method using the centrifugal force: the centrifugal mixed-powder method.

## 2. FGMs Fabricated by the Conventional Centrifugal Solid-Particle Method

The level of centrifugal force is characterized by the *G* number; here the *G* number is given by the following equation:

(1) $$G=\frac{\omega^{2}R}{g}$$

where *R* is the radius of the cast tube (in m) and ω is the mold spinning rate (in radians s ^-1^), and *g* is the acceleration due to gravity. The motion of solid particles in viscous liquid under centrifugal force can be determined by the Stokes's law. The terminal velocity is reached at a very early stage of the centrifugal casting method [8,13,14]. Therefore, the velocity of particles under centrifugal force, *dx/dt*, can be expressed as:

(2) $$\frac{dx}{dt}=\frac{|\rho_{p}-\rho_{m}|GgD_{p}^{2}}{18\eta }$$

where ρ_p_, ρ_m_, *D_p_* and η are density of particles, density of matrix, particle diameter and apparent viscosity of melt, respectively. Larger *dx/dt* value gives a steeper compositional gradient, and *vice versa*. From Equation (2), it is obvious that the compositional gradient formed by the centrifugal method is strongly affected by the particle size.

**Figure 1:**
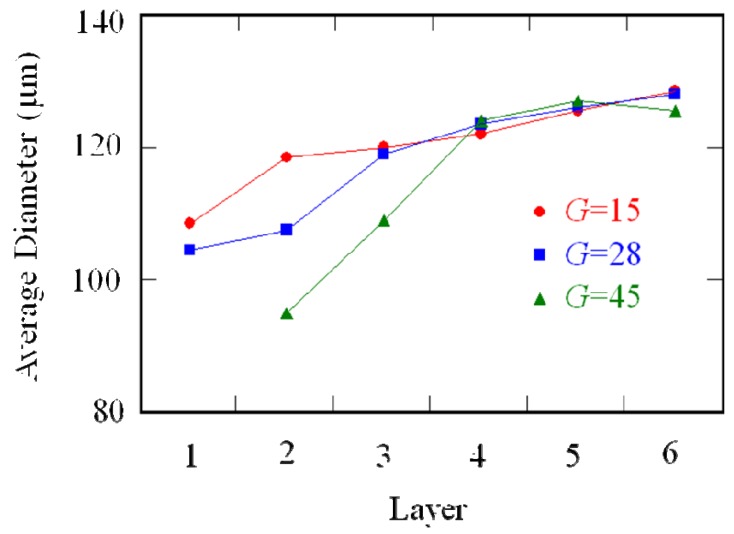
The average particle size of plaster/corundum model FGM fabricated by the centrifugal solid-particle method [13].

To study the particle size distributions in FGMs made by the centrifugal solid-particle method, plaster/corundum model FGM tubes containing five different particle sizes (the average particle sizes are 87 μm, 102.5 μm, 115 μm, 137 μm and 179.5 μm) were fabricated [13]. The mean volume fraction of particles is 16 vol %. The particle size distributions were directly determined by the extraction of the particles from the plaster matrix. The FGM tube was radially divided into six regions in equal width defined as layers 1 to 6 in sequence from the inner side, and the average particle size in each layer was calculated. Figure 1 shows the average particle size plotted against the radial position [13]. It is seen that the average particle size is gradually distributed in the FGMs, and the average particle size at outer region is greater than that in the inner region. Moreover, it is also found that the particle size gradient is increased with the increase of *G* number. It should be emphasized here that the particle size gradients are caused by the difference in velocity between larger particles and smaller particles. Therefore, the particle size strongly influences the compositional gradient in the FGM fabricated by the centrifugal solid-particle method.

**Figure 2:**
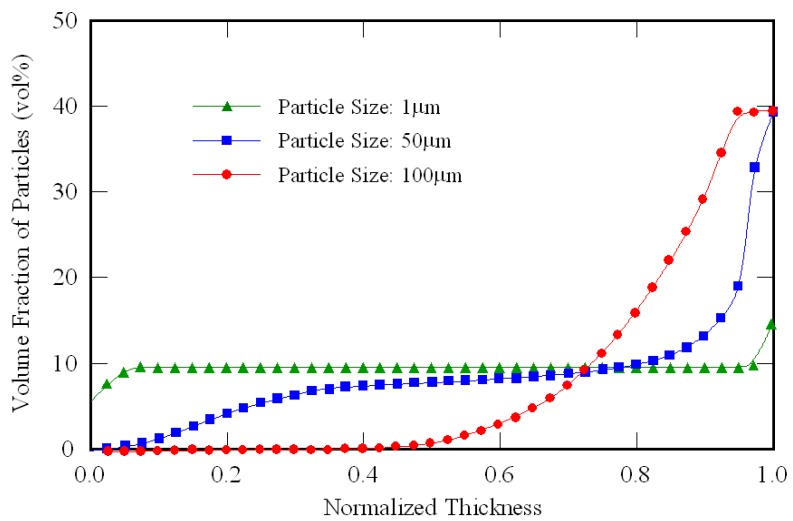
Particle distribution in an FGM fabricated by centrifugal solid-particle method [16].

Recently, many kinds of functional powders have been developed. One of such functional powders is TiO_2_ [15]. Because TiO_2_ powder has photocatalytic properties, it has been attracting attention for medical materials or construction materials. Since the photocatalytic TiO_2_ particles are of nano-sized diameter, the motion of TiO_2_ particles with different particle size in a molten Al under a centrifugal force is numerically modeled based on the Stokes’s law to study the effect of particle size on the compositional gradient. Figure 2 shows the effect of particle size on the compositional gradient in the Al/TiO_2_ FGM fabricated by the centrifugal solid-particle method [16]. Volume fraction of TiO_2_ and centrifugal force were fixed to be 10 vol % and *G* = 50, respectively. The abscissa represents the position of normalized thickness of the ring *i.e.*, 0.0 is the inner surface and 1.0 is the outer surface. As can be seen in the figure, TiO_2_ with 100 μm in diameter is gradually distributed in Al/TiO_2_ FGM, however, TiO_2_ particles with 1 μm in diameter were almost homogeneously distributed in the specimen. From these calculated results, it is concluded that the compositional gradient of nano-particle in the FGMs fabricated by the centrifugal solid-particle method is very small. Therefore, it is the difficult to fabricate an FGM containing nano-particles by the centrifugal solid-particle method.

## 3. Centrifugal Mixed-Powder Method for Gradual Distribution of Fine Particles

**Figure 3:**
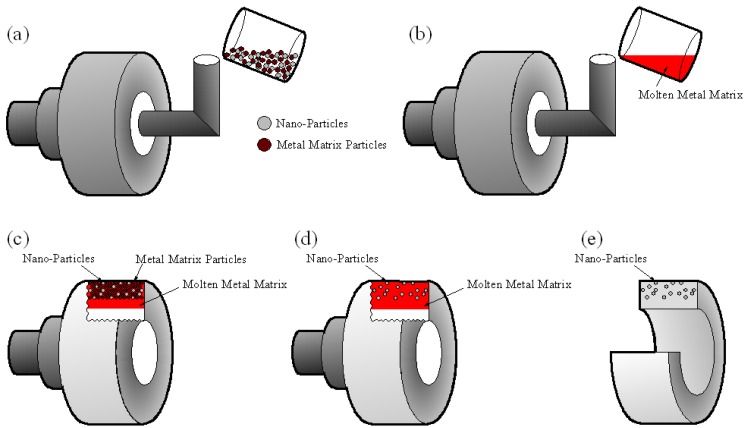
The schematic illustration showing the process of the centrifugal mixed-powder method.

With the above in mind, we are proposing a centrifugal mixed-powder method as a novel processing technique to fabricate a nano-particle distributed FGM. The experimental procedure of the centrifugal mixed-powder method is summarized in Figure 3. As a first step, a powder mixture of functional nano-particles and metal matrix particles is inserted into a spinning mold (Figure 3a). After that, a metal matrix ingot is melted and then the molten metal matrix is poured into the spinning mold with the powder mixture (Figure 3b). As a result, the molten metal matrix penetrates into the space between the particles due to the pressure exerted by the centrifugal force (Figure 3c). At the same time, the metal matrix powder is melted by the heat from molten matrix poured from the crucible (Figure 3d). Finally, an FGM ring with functional nano-particles distributed on its surface can be obtained (Figure 3e). Using this processing method, Cu/SiC and Al/TiO_2_ FGMs were fabricated, and these results are reviewed in the following two sections.

## 4. Cu/SiC FGM Fabricated by the Centrifugal Mixed-Powder Method

Cu-30vol%SiC and Cu-44vol%SiC powder mixtures were fabricated using pure Cu particles (99.9%, 1 mm and <45μm in diameter) and SiC powder. In order to investigate the effects of the SiC particle size on the microstructure of the FGM, three kinds of SiC powders with 150 μm, 50 μm and 500 nm in diameter were used. Using these powder mixtures, Cu/SiC FGM was fabricated by the centrifugal mixed-powder method using vertical-type centrifugal casting machine [17]. 

The applied centrifugal force was *G* = 100, and the spinning mold containing the powder mixture was heated up to 800 °C. A pure Cu ingot with purity of 99.9% was melted in a crucible with an induction furnace, and the molten Cu was poured into the spinning mold. After casting, the spinning mold was air-cooled. The fabricated cylindrical FGMs have the outer diameter of 40 mm, a length of 57 mm and thickness of about 10 mm. The details of the casting conditions are summarized in Table 1.

**Table 1:** Details of fabricated Cu/SiC FGMs.

Name of powder mixture	SiC powder (solid particle)		Pure Cu powder (Matrix)		Total weights of powder mixture and pure Cu ingot	Volume fraction of SiC in FGM surface
	Diameter	Weight	Diameter	Weight		
Specimen 1	150 μm	1.08 g		7.12 g	8.2 g/400 g	30 vol %
Specimen 2	50 μm	1.08 g	1 mm and	7.12 g		
Specimen 3	500 nm	1.08 g	45 μm under	7.12 g		
Specimen 4	50 μm	1.83 g		6.37 g		44 vol %

**Figure 4:**
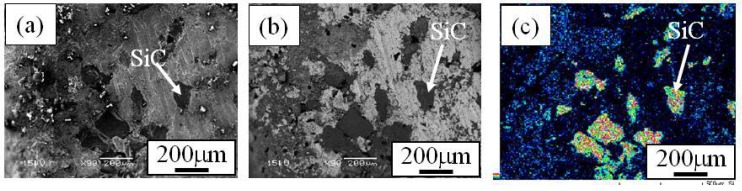
Microstructures on the surface of Specimens 1. (a) SE Image, (b) Compo Image and (c) Si distribution map by EDX. In a compo image, dark part shows SiC particle because material with lighter atomic weight is presented as darker image [17].

**Figure 5:**
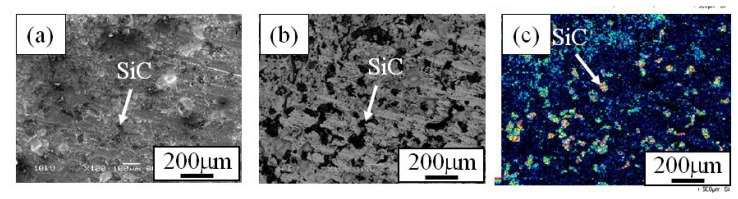
Microstructures on the surface of Specimens 2. (a) SE Image, (b) Compo Image and (c) Si distribution map by EDX [17].

**Figure 6:**
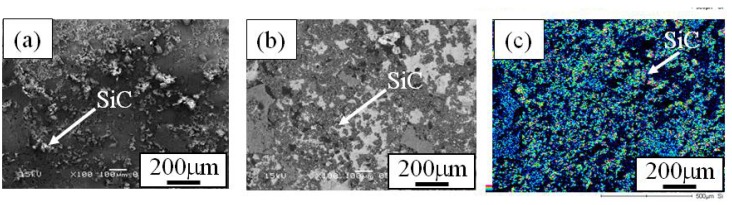
Microstructures on the surface of Specimens 3. (a) SE Image, (b) Compo Image and (c) Si distribution map by EDX [17].

When the Cu/SiC FGM cylinder was removed from the mold, no powders dropped out. Therefore, it is considered that all of the SiC particles in the powder mixture remained in the Cu/SiC FGM. A secondary electron image (SE image), a compositional image by backscatter electron (compo image) and a Si distribution map by EDX showing the microstructure on the outer surface of Specimen 1 are shown in Figures 4(a), (b) and (c), respectively [17]. On the other hand, results from Specimen 2 and Specimen 3 are shown in Figure 5 and Figure 6, respectively. SiC particles are observed on the outer surface of both the specimens. From Figures 4 to Figure 6, it is seen that SiC particles are successfully distributed on the surface of the FGMs by the centrifugal mixed-powder method. In all FGMs, the SiC particles are homogeneously distributed on the surface. Especially, Specimen 2 has denser distribution of fine SiC particles. 

**Figure 7:**
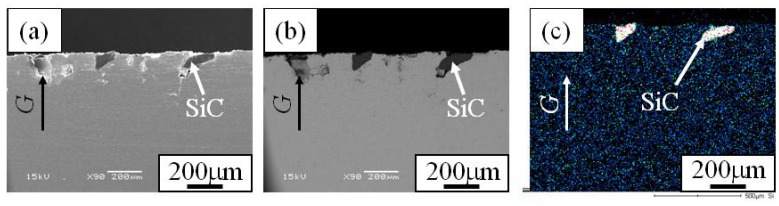
Microstructures of the cross-section of Specimens 1. (a) SE Image, (b) Compo Image and (c) Si distribution map by EDX [17].

**Figure 8:**
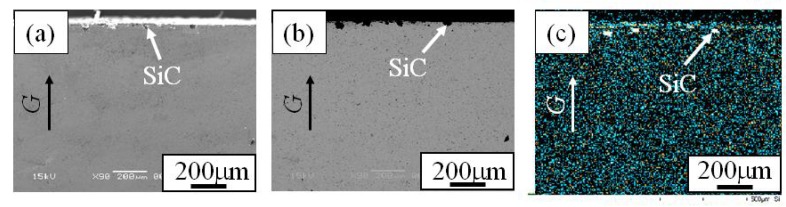
Microstructures of the cross-section of Specimens 2. (a) SE Image, (b) Compo Image and (c) Si distribution map by EDX [17].

**Figure 9:**
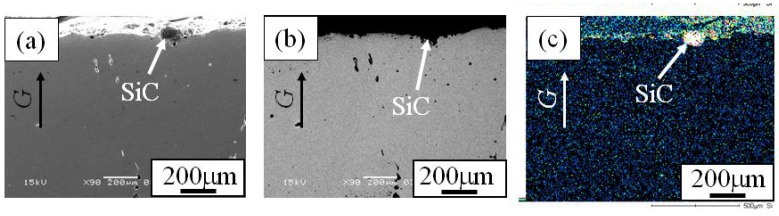
Microstructures of the cross-section of Specimens 3. (a) SE Image, (b) Compo Image and (c) Si distribution map by EDX [17].

In order to investigate the distribution of SiC particles inside the FGM, microstructures on the cross-sectional plane of the FGMs were observed. Figures 7 (a), (b) and (c) are the SE image, compo image and Si distribution map of the cross-sectional plane of Specimen 1, respectively. Similarly, Figure 8 and Figure 9 show microstructure of the cross-sectional planes of Specimen 2 and Specimen 3, respectively. As shown in Figure 7, Figure 8 and Figure 9, the SiC particles of both specimens are embedded in Cu matrix. Moreover, SiC particles are distributed near the surface of the FGM and no SiC particles are observed in the interior and inner parts of the FGMs. The density of SiC is much smaller than that of molten Cu matrix (SiC; 3.217 Mg/m^3^, Cu; 8.960 Mg/m^3^). Regardless of such a small SiC density, the SiC particles are distributed only near the surface of the FGM. This means that the movement of the solid particles in the powder mixture by the centrifugal mixed-powder method is relatively small. Thus, it is expected that gradual distribution of the solid particle is formed by controlling its volume fraction in powder mixture.

**Figure 10:**
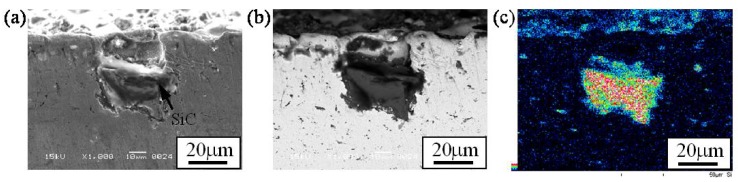
Microstructure around a SiC particle in Specimen 2. (a) SE Image, (b) Compo Image and (c) Si distribution map by EDX [17].

Figures 10 (a), (b) and (c) are an SE image, compo image and Si distribution map showing the microstructure around a SiC particle in Specimen 2, respectively. No intermediate phase is observed on the interface between SiC and the Cu matrix. From this micrograph, it is seen that SiC particles are just physically fixed by the Cu matrix [17]. Because of it, if a SiC particle is largely cropped out from surface, the SiC particle would have dropped out. As will be described later, it is better to generate the reaction such as SHS between matrix and solid particle to fix solid particles tightly.

**Figure 11:**
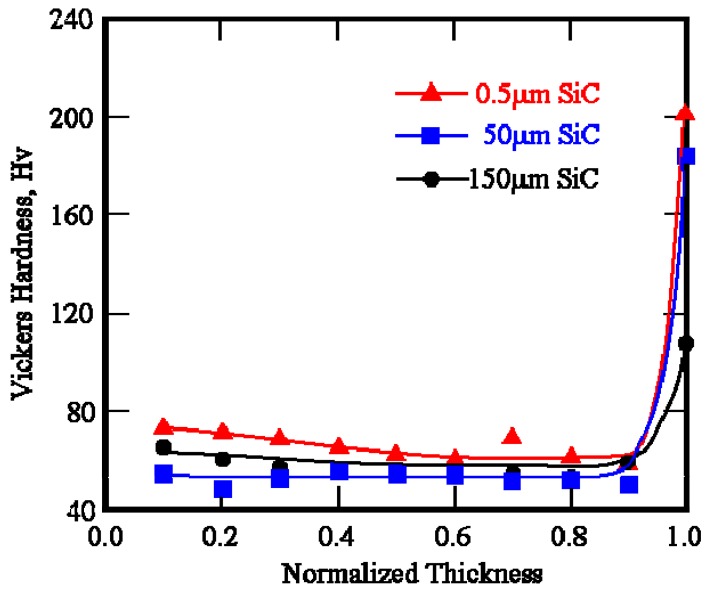
Vickers hardness of Specimens 1, 2 and 3 as a function of normalized thickness [17].

Vickers hardness distributions of Cu/SiC FGMs as a function of normalized thickness are shown in Figure 11. Load and holding time for the Vickers hardness tests were 1.98 N and 30 s, respectively. As shown in Figure 11, Vickers hardness distribution inside Cu/SiC FGMs are constant with the value of about Hv = 55. On the other hand, higher Vickers hardness values have been found on the surface of the Cu/SiC FGMs. Specimen 3 with smaller SiC particles has higher hardness on the surface of the FGM than Specimen 1 with larger SiC particles. Smaller particles can improve the hardness on the surface effectively rather than larger SiC particles under the same volume fraction. In this way, the mechanical properties of nano-particle dispersed FGMs are superior to those of conventional FGMs. This is the motivation of fabrication of the FGM containing nano-TiO_2_ particles by the centrifugal mixed-powder method, and will be described in the next section.

**Figure 12:**
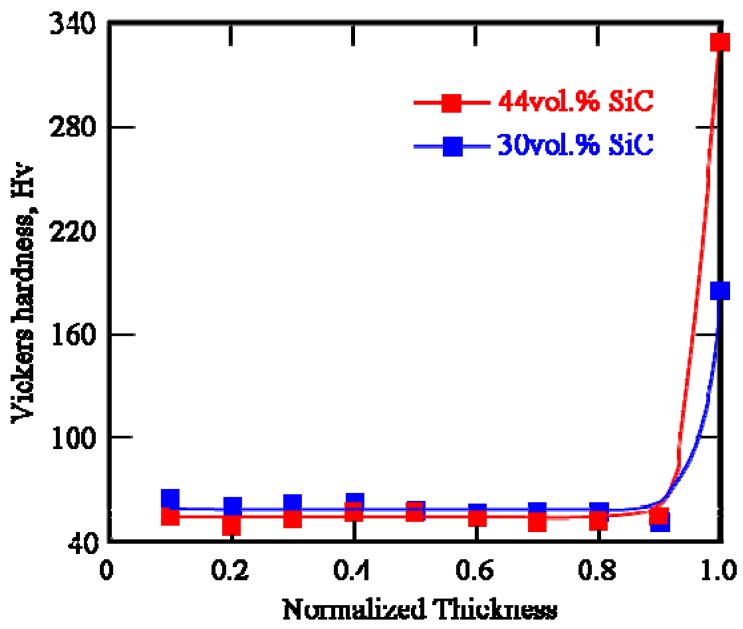
Vickers hardness of Specimens 2 and 4 as a function of normalized thickness.

To study the effect of particle volume fraction on the mechanical properties, Specimen 4, which has 44 vol % SiC at the ring outer region, was fabricated. The Vickers hardness distribution of Specimen 4 (Cu/44vol%SiC FGM) is shown in Figure 12. The data from Specimen 2 (Cu/30vol%SiC FGM) are also shown in this figure for comparison. As can be seen, higher hardness was found for Specimen 4 with a higher volume fraction of reinforcements due to well-known dispersion-hardened effect. In a metal matrix composite (MMC) subjected to a temperature change *ΔT*, the mismatch of the coefficients of thermal expansion between the matrix and reinforcements causes a residual stress. When the residual stress becomes larger than a certain value, dislocations are punched out into the matrix to relax the stress. Dislocations left in the plastic domains after punching strongly affect the mechanical properties of MMCs. Since the amount of punched out dislocations in MMCs is related to the volume fraction of reinforcements, the hardness of MMCs also depends on the volume fraction of reinforcements [18].

## 5. Al/TiO_2_ FGM Fabricated by the Centrifugal Mixed-Powder Method [16]

The primary particle-diameters of the Al and the TiO_2_ powders were 93 μm and ~500 nm, respectively. These particles were mixed. The crystal structure of the TiO_2_ used was anatase structure. To investigate the effects of the volume fraction of TiO_2_ particles in powder mixtures on the particle distribution of TiO_2_ in Al/TiO_2_ FGM, mixed-powders with different TiO_2_ volume fractions were prepared, *i.e.*, Al-10vol%TiO_2_ and Al-30vol%TiO_2_. Table 3 shows the details of fabricated Al-TiO_2_ powder mixtures.

Al/TiO_2_ FGMs were fabricated by the centrifugal mixed-powder method using these powder mixtures. A horizontal-type centrifugal casting machine was conducted for this experiment [16]. The applied centrifugal force was *G* = 80. After casting, the spinning mold was air-cooled. The fabricated FGM cylinder has the outer diameter of 90mm, the length of 30mm and thickness of about 20mm. The details of casting conditions are summarized in Table 4.

**Table 3:** Powder mixtures prepared for Al/TiO_2_ FGMs.

Name of powder mixture	TiO_2_ powder (Solid particle)		Pure Al powder (Matrix)		Total weight of powder mixture
	Diameter	Weight	Diameter	Weight	
Mixture A	~500 nm	0.86 g	93 μm	5.34 g	6.2 g
Mixture B	~500 nm	3.06 g	93 μm	5.20 g	8.26 g

**Table 4:** Casting conditions for Al/TiO_2_ FGMs.

Name of specimen	Used Mixed-powder	Temperature		Weight of molten Al
		Processing temperature	Mold temperature	
Specimen A	Mixture A	750 °C	900 °C	443.4 g
Specimen B	Mixture B	660 °C	650 °C	419.3 g

**Figure 13:**
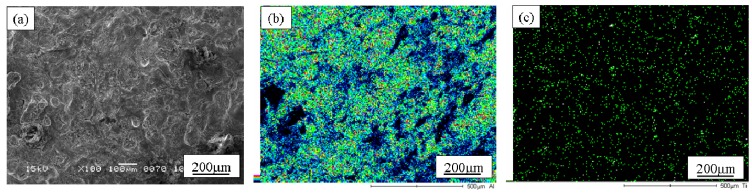
SEM microstructure, Al and Ti distribution maps on outer surface of the Specimen A [16].

**Figure 14:**
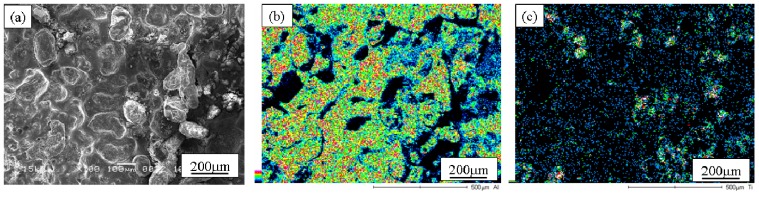
SEM microstructure, Al and Ti distribution maps on outer surface of the Specimen B [16].

Figures 13 (a), (b) and (c) show the SEM microstructure, Al and Ti distribution maps on outer surface of the Specimen A, respectively [16]. SEM microstructure, Al and Ti distribution maps on the outer surface of the Specimen B are shown in Figures 14 (a), (b) and (c), respectively. As can be seen in Figure 13 and Figure 14, TiO_2_ particles are observed on the outer surface of both the Al/TiO_2_ FGMs. Although it is difficult to distribute nano-particles gradually in the FGM cylinder by the centrifugal solid-particle method, TiO_2_ nano-particles are successfully distributed on the outer surface by the centrifugal mixed-powder method. Comparing between the microstructures of Specimens A and B, TiO_2_ nano-particles were more clearly observed on the outer surface of Specimen B rather than in Specimen A. 

**Figure 15:**
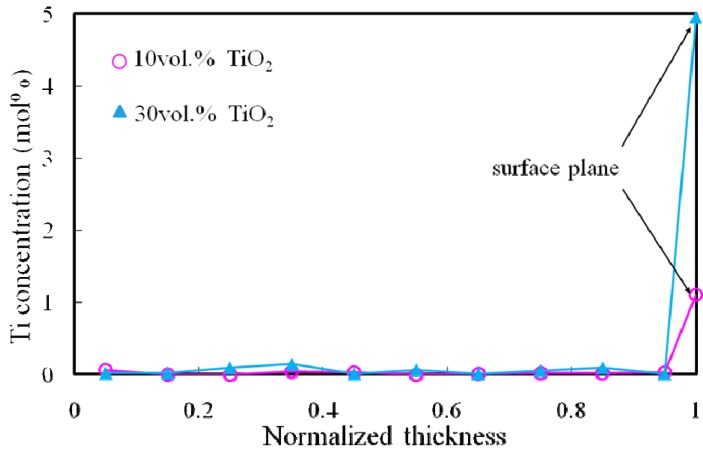
Distributions of Ti concentration in Al/TiO_2_ FGMs.

Figure 15 shows the Ti concentration of both Al/TiO_2_ FGMs as a function of normalized thickness [16]. From this graph, Ti concentration in both Al/TiO_2_ FGMs is almost constant from the normalized thickness of 0 to 0.95, and no Ti is detected inside the Al/TiO_2_ FGMs. However, on the surface of the FGM rings, Ti is detected. From these results, it is found that TiO_2_ nano-particles are distributed only on surface of Al/TiO_2_ FGMs. Moreover, Specimen B has higher Ti concentration than Specimen A. In this way, TiO_2_ nano-particle distribution on the surface of ring may be controlled by the volume fraction of TiO_2_ nano-particle in powder mixture.

**Figure 16:**
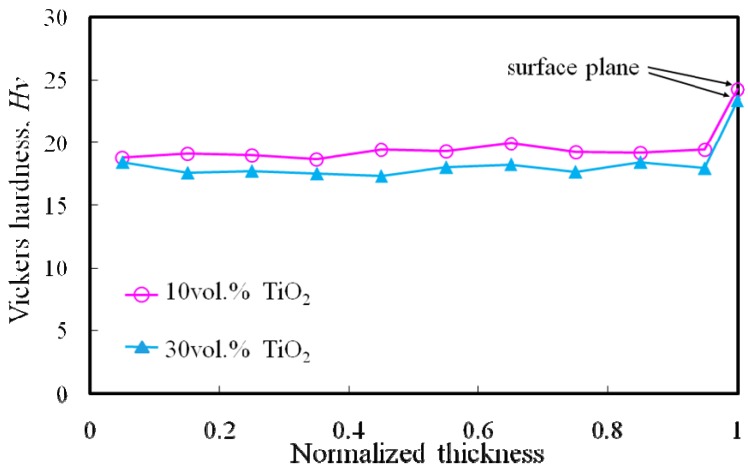
Distribution of the Vickers hardness in Al/TiO_2_ FGMs.

Figure 16 shows Vickers hardness distributions of both Al/TiO_2_ FGMs as a function of normalized thickness [16]. Load and holding time for the Vickers hardness tests were 3.67 N and 30 s, respectively. As shown in Figure 16, Vickers hardness value inside Al/TiO_2_ FGMs is constant with about Hv = 19, regardless of the volume fraction of TiO_2_ in the powder mixture. This hardness is almost same as the hardness of pure Al [19]. On the other hand, the Vickers hardness on the surface is the highest, as shown in Figure 16. Generally, the dispersion of such fine TiO_2_ particles improves the strength of the soft matrix by dispersion hardening. Therefore, this hardness improvement on the surface of the Al/TiO_2_ FGM is caused by distribution of TiO_2_ nano-particles.

## 6. Merit of the Centrifugal Mixed-Powder Method

From the results described in this article, it is clear that centrifugal mixed-powder method is an effective method for fabrication of FGMs containing nano-particles. If such nano-particles are distributed by the centrifugal mixed-powder method, the fabrication of novel FGMs and their new applications can be expected. However, in order to apply this fabrication technique practically, it is important to control the nano-particle distribution in an FGM. Therefore, the establishment of ways of controlling the nano-particle distribution in the FGM is a topic of our future work.

Recently, the processing technique of the FGM using the SHS technique has been proposed [20,21,22]. If such a SHS reaction is occurred between solid particles and the matrix in the powder mixture, the solid particles on the surface of the FGM can be fixed tightly. Moreover, such tight fixation of the solid particle can be expected to improve the strength and the wear property on the surface of FGM.

Carbon fiber reinforced plastic (CFRP) is a very strong and light composite material. Due to its high ratio of strength to weight, it has many applications in aerospace and automotive fields, as well as in sailboats, and notably in modern bicycles and motorcycles. Improved manufacturing techniques are reducing the costs and time to manufacture. Although CFRP can be manufactured to near-net shape, post processing operations such as machining are not entirely avoidable to create some features, especially holes. In fact, drilling is the most common post-processing operation performed on CFRP. When holes are produced on CFRP, special attention should be focused on as the machining parameters influencing the damage. Therefore, the high-precision processing technology for CFRP is strongly required.

A new technique, drilling of CFRP by gyro-driving grindstone, is developed by Gifu Prefectural Research Institute for Machinery and Materials [23], where the grindstone is a tool used for grinding or sharpening tools. Using this technique, one can obtain relatively large holes without any burr and delamination of the CFRP. There is a need to develop the metal-bonded abrasive tool with nano-particle, if the size of holes become smaller and smaller and high precision processing technology for CFRP is required. In this case, the concept of FGMs can be applied, since the macroscopic interface between particle-reinforced region and matrix region must be eliminated. With the above in mind, we are developing nano-diamond reinforced functionally graded grindstone by a centrifugal mixed-powder method and high-precision processing technology for CFRP by using the functionally graded grindstone.

## 7. Summary

A novel fabrication method, the centrifugal mixed-powder method, by which we can obtain FGMs containing nano-particles, has been proposed. From the obtained results, it is found that the centrifugal mixed-powder method can disperse nano-particles only on the surface of FGMs. Moreover, the distribution of nano-particles in the FGM has no dependence on density difference between matrix and particle. At the same time, hardness on the surface of FGM fabricated by centrifugal mixed-powder method is improved by dispersion of nano-particles. Finally, it is concluded that the centrifugal mixed-powder method is an effective method for fabrication of FGMs containing nano-particles.

## References

[1] Suresh S., Mortensen A. (1998). Fundamentals of Functionally Graded Materials, Processing and Thermomechanical Behaviour of Graded Metals and Metal-Ceramic Composites.

[2] Miyamoto Y., Kaysser W.A., Rabin B.H., Kawasaki A., Ford R.G. (1999). Functionally Graded Materials: Design, Processing and Applications.

[3] Uemura S., Noda Y., Shinohara Y., Watanabe Y. (2009). Development and Technology of Functionally Graded Materials.

[4] Dumont A.-L., Bonnet J.-P., Chartier T., Ferreira J.M.F. (2001). MoSi_2_/Al_2_O_3_ FGM: Elaboration by Tape Casting and SHS. J. Euro. Cer. Soc..

[5] Cirakoglu M., Bhaduri S., Bhaduri S.B. (2002). Combustion Synthesis Processing of Functionally Graded Materials in the Ti–B Binary System. J. Alloys Compd..

[6] Royer A., Vasseur S., Stefanescu D.M. (1988). Horizontal Centrifugal. Casting, Metals Handbook.

[7] Fukui Y. (1991). Fundamental investigation of functionally gradient material manufacturing system using centrifugal force. JSME Inst. J. Series III.

[8] Watanabe Y., Yamanaka N., Fukui Y. (1998). Control of Composition Gradient in a Metal­Ceramic Functionally Graded Material Manufactured by the Centrifugal Method. Composites Part A.

[9] Watanabe Y., Yamanaka N., Fukui Y. (2000). Microstructures and Mechanical Properties of Functionally Graded Materials Fabricated by a Centrifugal Method. Rec. Res. Develop. Metall. Mater. Sci..

[10] Watanabe Y, Oike S. (2005). Formation Mechanism of Graded Composition in Al-Al_2_Cu Functionally Graded Materials Fabricated by a Centrifugal *in situ* Method. Acta Mater..

[11] Watanabe Y., Kim I.-S., Fukui Y. (2005). Microstructures of Functionally Graded Materials Fabricated by Centrifugal Solid-Particle and *in situ* Methods. Metal. Mater. Int..

[12] Watanabe Y., Sato H., Fukui Y. (2008). Wear Properties of Intermetallic Compound Reinforced Functionally Graded Materials Fabricated by Centrifugal Solid-particle and *in situ* Methods. J. Solid Mech. Mater. Eng..

[13] Watanabe Y., Kawamoto K., Matsuda K. (2002). Particle Size Distributions of Functionally Graded Materials Fabricated by Centrifugal Solid-Particle Method. Comp. Sci. Tech..

[14] Kang C.G., Rohatgi P.K. (1996). Transient Thermal Analysis of Solidification in a Centrifugal Casting for Composite Materials Containing Particle Segregation. Metall. Mater. Trans. B.

[15] Lee J.E., Oh S.-M., Park D.-W. (2004). Synthesis of Nano-sized Al Doped TiO_2_ Powders Using Thermal Plasma. Thin Solid Films.

[16] Inaguma Y., Sato H., Watanabe Y. (2010). Fabrication of Al-based FGM Containing TiO_2_ Nano-Particles by a Centrifugal Mixed-Powder Method. Mater. Sci. Forum.

[17] Sato H., Inaguma Y., Watanabe Y. (2010). Fabrication of Cu-based Functionally Graded Materials Dispersing Fine SiC Particles by a Centrifugal Mixed-Powder Method. Mater. Sci. Forum.

[18] Watanabe Y., Yamanaka N., Oya-Seimiya Y., Fukui Y. (2001). Micro-hardness Measurements to Evaluate Composition Gradient by in Metal-Based Functionally Graded Materials. Z. Metallkd..

[19] Sato H., Ota K., Zhang Z., Tsuzaki K, Watanabe Y. (2006). Grain Refinement Performance of Aluminum Cast-Alloy by Deformed Al-Al3Ti Alloy Refiner. Mater. Sci. Forum.

[20] Stangle G.C., Miyamoto Y. (1995). FGM Fabricated by Combustion Synthesis. MRS Bull..

[21] Lin J.S., Miyamoto Y., Tanihata K., Yamamoto M., Tanaka R. (1998). Toughening Effects of WC/Co Particles and Compressive Surface Stress on (Al_2_O_3_–WC/Co)/TiC/Ni Graded Materials. J. Mater. Sci..

[22] Watanabe Y., Watanabe S., Matsuura K. (2004). Nickel-aluminides/Steel Clad Pipe Fabricated by Reactive Centrifugal Casting Method from Liquid Aluminum and Solid Nickel. Metall. Mater. Trans. A.

[23] Watanabe Y. Development of Nanocomposite Based on Interface Engineering. ISPlasma2009, First International Symposium on Advanced Plasma Science and its Applications.

